# Role of Wnt-signaling inhibitors DKK-1 and sclerostin in bone fragility associated with Turner syndrome

**DOI:** 10.1007/s40618-022-01760-3

**Published:** 2022-03-02

**Authors:** M. Chiarito, L. Piacente, N. Chaoul, P. Pontrelli, G. D’Amato, A. Grandone, G. Russo, M. E. Street, M. G. Wasniewska, G. Brunetti, M. F. Faienza

**Affiliations:** 1 Department of Biomedical Sciences and Human Oncology, Pediatric Unit, University of Bari “A. Moro”, Bari, Italy; 2 Department of Biomedical Sciences and Human Oncology, University of Bari “A. Moro”, Bari, Italy; 3 Department of Emergency and Organ Transplantation, Division of Nephrology, University of Bari “A. Moro”, Bari, Italy; 4 Neonatal Intensive Care Unit, “Di Venere” Hospital, Bari, Italy; 5 Department of Woman, Child and General and Specialized Surgery, University of Campania Luigi Vanvitelli, Naples, Italy; 6 Department of Pediatrics, IRCCS San Raffaele Hospital, Milan, Italy; 7 Department of Mother and Child, Azienda USL-IRCCS Di Reggio Emilia, Reggio Emilia, Italy; 8 Pediatric Unit, Department of Human Pathology in Adulthood and Childhood, University of Messina, Messina, Italy; 9 Department of Biosciences, Biotechnologies and Biopharmaceutics, University “A. Moro” of Bari, Bari, Italy

**Keywords:** Turner’s syndrome, Skeletal fragility, DKK-1, Sclerostin, RANKL

## Abstract

**Purpose:**

Girls affected with Turner syndrome (TS) present with low bone mineral density (BMD) and osteopenia/osteoporosis. Thus, they have an increased risk to develop fractures compared to normal population. The aim of this study was to deepen the pathophysiology of skeletal fragility in TS subjects by evaluating the serum levels of Dickkopf-1 (DKK-1) and sclerostin, main regulators of bone mass, as well as the percentage of circulating osteoblast precursors (OCPs).

**Methods:**

Thirty-four TS girls and 24 controls were recruited. All subjects underwent anthropometric measures (height, weight, body mass index-BMI). A peripheral venous blood sample was collected to determine serum levels of active intact parathyroid hormone (PTH), 25-OH vitamin D, calcium, phosphorus, bone alkaline phosphatase (bALP), osteocalcin, sclerostin, DKK-1, RANKL and OPG. OCPs were detected by flow cytometry. In TS subjects bone mineralization was measured at lumbar spine by dual energy X-ray absorptiometry (DXA).

**Results:**

bALP, 25-OH Vitamin D, and osteocalcin levels were significant lower in TS subjects than in the controls. Statistically significant higher levels of sclerostin, DKK-1 and RANKL were measured in patients compared with the controls. The percentage of OCPs did not show significant differences between patients and controls. Sclerostin and DKK-1 levels were related with anthropometric parameters, bone metabolism markers, HRT, rhGH therapy, RANKL and lumbar BMAD-Z-score.

**Conclusion:**

TS patients showed higher levels of sclerostin and DKK-1 than controls which can be related to HRT, and to reduced bone formation markers as well as the increased bone resorption activity.

## Introduction

Turner syndrome (TS) is a chromosomal disorder resulting from total or partial loss of one of the X chromosomes in phenotypically female patients. Clinical features depend on the karyotype, and usually include short stature, hypergonadotropic hypogonadism due to ovarian dysgenesis, cardiac defects and neurocognitive impairment [1]. About 45% of TS women present with low bone mineral density (BMD) and osteopenia/osteoporosis, with an earlier onset than that observed for postmenopausal osteoporosis [2]. Thus, TS subjects have an increased risk of about 25% of developing fractures compared to the normal population, particularly during infancy and after 45 years of age [3]. Typical features in TS subjects are the reduction of cortical BMD and cortical thickness, while trabecular bone is generally preserved [4, 5].

Bone health depend on the correct balance between deposition of new bone by osteoblasts (OBs) and resorption from osteoclasts (OCs); the former is mainly regulated by Wnt/*β*-catenin pathway, the latter by the receptor activator of NF-kB (RANK)/RANK ligand (RANKL)/osteoprotegerin (OPG) axis [6, 7]. Multiple factors are responsible for bone impairment in people with TS, as well as estrogen deficiency, haploinsufficiency of X chromosome genes, alterations in vitamin D metabolism, and comorbidities frequently associated with TS and other chromosomopathies, such as autoimmune diseases [8–10]. In a previous study we demonstrated an enhanced osteoclastogenesis due to high levels of circulating FSH in TS girls before hormonal replacement therapy (HRT). FSH acts both directly by binding its specific receptor on the OCs, and indirectly by enhancing the production of TNF-*α*, a pro-osteoclastogenic cytokine, from bone marrow macrophages and T-cells. Conversely, the increased osteoclastogenesis observed in TS girls and women on HRT is due to high RANKL levels [11]. RANKL and OPG levels are modulated by canonical Wnt/*β*-catenin inhibitors, such as Dickkopf-1 (DKK-1) and sclerostin, which are the main regulators of bone mass [12]. The role of these two inhibitors has been demonstrated in several congenital and acquired diseases [13]. In particular, sclerostin and DKK-1 inhibit the differentiation and activity of OBs, decrease OPG levels, and increase RANKL expression in the cells of osteoblastic lineage, thereby shifting the OPG/RANKL ratio in favor of bone resorption [12, 14]. There are no data about the activity of OBs, the bone forming cells, in TS patients. Due to the difficulty to obtain bone biopsy from patients to isolate OBs, our research has been directed to the circulating osteoblast precursors (OCPs), which can be detected by flow cytometry in the peripheral human blood, and are related to osteoblast markers, such as alkaline phosphatase and osteocalcin [15, 16]. In postmenopausal osteoporosis, a decreased percentage of OCPs has been reported [17]. Furthermore, an involvement of this cell population in skeletal growth and fracture repair has been demonstrated [18].

The aim of this study was to deepen the pathophysiology of skeletal fragility in TS subjects by evaluating the serum levels of DKK-1 and sclerostin, as markers of possible impairment of cells of the osteoblastic lineage, as well as the percentage of OCPs.

## Patients and methods

### Subjects

For this study, we recruited 34 girls affected by TS who referred to 5 Italian Pediatric Endocrinology Centers.

According to the karyotype, 15 out of the 34 TS patients had X monosomy (45,X), 10 had 45,X/46,XX mosaicism, 6 had isochromosome of the Xq, 1 had isochromosome of the Xp, 2 had ring-X chromosome. Twenty girls out of the 34 (58.8%) aged 12–18 years were on HRT. Regarding the 14 patients with TS who were not on HRT, 12 were the youngest in the study population, and 2 patients with 45,X/46,XX mosaicism had spontaneous sexual development.

For the puberty induction the patients with a chronological age of 12 years received transdermal administration of ethinyl estradiol at the dosage of 100 ng per kilogram per day for 9–12 months, monitoring the response of breast development and endometrial thickness by ultrasonography. Thirteen TS patients out of the 34 (38.2%) had discontinued recombinant growth hormone (rhGH) therapy as the growth rate was less than < 2 cm/year. None of them had history of fractures.

Inclusion criteria were a karyotype diagnosis of TS (without Y-chromosome material), and a normal thyroid function for at least 3 months in patients with hypothyroidism. Exclusion criteria included use of corticosteroids, presence of chronic disease, calcium and/or vitamin supplementation. As control group we recruited 24 age-matched girls who referred to our hospital for minor surgery or electrocardiographic screening. The study size was determined considering a power of our tests on 25-OH vitamin D and sclerostin levels between 0.9 and 1.

The work was approved by Ethical Committee of the Policlinico of Bari as the coordinating center, and by the ethical committees of the participating centers. Written informed consent was obtained from the patients’ parents or guardians, and from the patients when appropriate. The study was performed according to the Declaration of Helsinki.

### Auxological and biochemical assessments

TS girls and controls underwent anthropometric measures. Height, weight, and Body mass Index (BMI) were converted to age specific standard deviation scores (SDS) on the basis of reference data reported on growth calculator 4 [19]. Pubertal development was evaluated according to Tanner [20]. From patients and controls, after an overnight fast, venous peripheral blood samples were taken in opportune tubes for serum separation as well as in EDTA tubes for flow cytometry. For serum separation, the samples were opportunely centrifuged and immediately frozen at − 80 °C until the determination was performed. Serum active intact parathyroid hormone (PTH) and 25-OH vitamin D were measured by immunological tests based on the principle of chemiluminescence using commercial kits (Liaison assay; DiaSorin, Stillwater, MN). Osteocalcin serum concentration was measured by Enzyme Immuno Assay, using commercial kit (IBL, International, Germany). Calcium, phosphorus, and bone alkaline phosphatase (bALP) serum concentrations were measured by nephelometric method. DKK-1 (R&D Systems, Minneapolis, MN), sclerostin, RANKL and OPG were also assessed by ELISA (Biomedica Medizinprodukte, GmbH & Co KG, Vienna, Austria). For the assessment of the above cytokines, all samples were centralized to a single laboratory. The specific characteristics of each ELISA kits are the following: DKK-1: assay range 31.3–2000 pg/mL with intra-assay coefficient of variation (CV) ≤ 4.2%, and inter-assay CV ≤ 7.6%; Sclerostin: assay range 0–240 pmol/l with intra-assay CV ≤ 7% and inter-assay CV ≤ 10%; RANKL: assay range 0–2 pmol/l with intra-assay CV ≤ 4% and inter-assay CV ≤ 3%; OPG: assay range 0–20 pmol/l with intra-assay CV ≤ 3% and inter-assay CV ≤ 5%.

### Bone mineral measurements

Bone mineralization was measured at lumbar spine L2–L4 by dual energy X-ray absorptiometry (DEXA) with the equipment present at each center (ACN Unigamma X-Ray Plus; L’ACN Scientific Laboratories; Hologic DiscoveryWi; Lunar iDXA, GE Healthcare) and converted to SD scores (*Z*-scores) in relation to age and sex-matched normal population. Height also was measured at the time of BMD evaluation. To minimize the effect of body size on areal BMD results at the lumbar spine, a validateLd transformation was made of the DXA data to calculate a volumetric density [bone mineral apparent density (BMAD)]. The *Z*-score values for height and for BMAD were calculated by subtracting the corresponding age- and sex-matched values and dividing by the corresponding standard deviation.

### Flow cytometry

The peripheral blood mononuclear cells (PBMCs) were isolated from the peripheral blood of patients and controls by gradient centrifugation using Ficoll Paque (from GE healthcare). PBMCs were immediately analyzed by flow cytometry or frozen at − 80 °C until use. The isolated PBMCs were stained with fluorochrome-conjugated antibodies for the detection of osteoblast precursors: PE-Cy7-coupled anti-CD34 (from Beckman Coutler, Milan, Italy), PE-coupled anti-osteocalcin and APC-coupled anti-alkaline phosphatase (both from R&D Systems). PBMCs were incubated for 20 min with the antibodies in the dark at 4 °C and washed twice. Stained cells were then acquired on a Navios cytometer (Beckman Coulter) and analyzed using the FlowJo software (Tree Star Inc., USA). Detection and analysis were centralized to a single laboratory.

### Statistical analyses

For statistical analyses, the Statistical Package for the Social Sciences for Windows, version 27.0 (SPSS Inc., Chicago, IL) was utilized. Results are reported as means ± standard deviation. The absence of therapy was set to 0, whereas the occurrence of therapy was set to 1. To determine the normality of the distribution of the studied parameters, the Kolmogorov–Smirnov test was used. In parameters with normal distribution, mean values were compared using the unpaired Student *t* test, whereas linear correlations were evaluated with the Pearson correlation coefficient. Whereas, in parameters with skewed distribution, significance was assessed with the Mann–Whitney test and Spearman correlation coefficient. Comparisons among groups were performed by one-way ANOVA. Finally, multiple regression analyses were performed to identify predictors of DKK-1 and sclerostin levels. The limit of statistical significance was set at 0.05.

## Results

### Bone status in TS patients

**Fig. 1:**
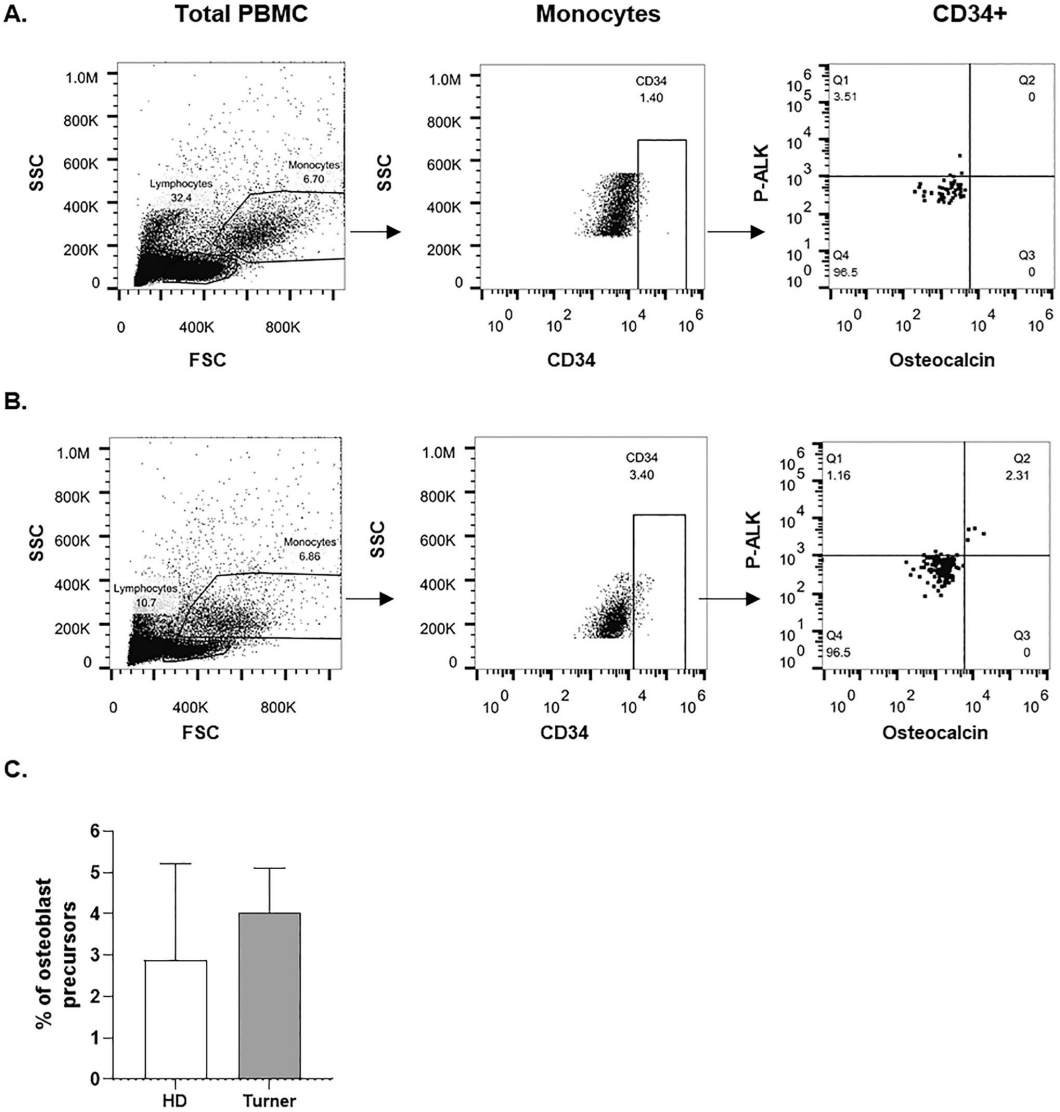
PBMCs were isolated from peripheral blood by gradient centrifugation and stained with anti-CD34, anti-osteocalcin and anti-alkaline phosphatase. Cells were then analyzed by flow cytometry according to the following gating strategy: monocytes were gated based on the FSC and the SSC, then osteoblast precursors were selected based on the expression of CD34. Finally, the expression of osteocalcin and alkaline phosphatase were analyzed on CD34^+^ cells. The dot plots shown in (**A**) are representative of healthy donors, those shown in (**B**) are representative of Turner patients. **C** Frequencies of osteoblast precursors in healthy donors (HD; clear histogram) and turner patients (grey histogram) are shown. Results are expressed as mean ± SEM from cumulative results

Table 1 shows patients’ baseline characteristics as well as biochemical and instrumental parameters of bone metabolism. TS subjects displayed significant reduced height-SDS and increased BMI–SDS compared with the controls. Interestingly, patients showed reduced levels of bone formation markers, such as bALP, 25-OH Vitamin D, and osteocalcin with respect to the controls. Lumbar spine BMAD-Z-score fell in the physiological range for 14 TS patients (0.21 ± 0.86), in the osteopenic range for 13 TS subjects (− 1.64 ± 0.43), and in the osteoporotic range (− 2.79 ± 0.38) for the remaining 7 TS patients. Consistent with these results, statistically significant higher levels of sclerostin and DKK-1, inhibitors of osteoblast activity, were measured in patients compared with the controls (Table 1). We also confirmed the higher levels of RANKL in TS subjects than controls, and the no statistically significant levels of OPG compared with the controls. These results prompted us to measure by flow cytometry the percentage of OCPs through the evaluation of the expression of CD34^+^, bALP and osteocalcin in the monocyte compartment. No statistically significant differences were detected between controls and patients (2.89 ± 2.32 vs 4.02 ± 1.08) (Fig. 1).

**Table 1:** Baseline characteristics, biochemical and instrumental data of TS patients and controls

	Turner (*n* = 34)	Controls (*n* = 24)	*p* value
Age (yrs)	13.29 ± 3.58	11.23 ± 3.80	ns
Karyotype (monosomy/mosaicism)	15/19	–	–
Height-SDS	− 2.22 ± 0.90	− 0.23 ± 0.99	˂0.001
Weight-SDS	− 0.44 ± 1.13	0.05 ± 0.86	ns
BMI–SDS	0.75 ± 1.15	0.10 ± 0.91	˂0.01
Tanner stage (I/II/III/IV/V)	14/3/3/3/11	11/2/5/2/4	ns
HRT (yes/no)	20/14	–	–
rhGH therapy (yes/no)	21/13	–	–
IGF-1 (ng/ml)	336 ± 164	–	–
Ca, mg/dl	9.68 ± 0.36	9.74 ± 0.30	ns
P, mg/dl	4.44 ± 0.68	4.54 ± 0.43	ns
bALP, μg/L	29.80 ± 14.66	86.30 ± 28.11	≤ 0.001
25-OH vitamin D, ng/ml	20.08 ± 6.31	27.54 ± 6.53	≤ 0.001
PTH, pg/ml	27.92 ± 14.49	23.93 ± 7.99	ns
Osteocalcin, ng/ml	68.68 ± 46.93	102.24 ± 27.28	≤ 0.001
Lumbar BAMD-*Z*-score	− 1.25 ± 1.34	–	–
Sclerostin, pmol/l	20.47 ± 5.81	14.27 ± 3.38	0.001
DKK-1, pg/ml	4341 ± 1085	2607 ± 345	≤ 0.001
RANK-L, pmol/l	0.32 ± 0.16	0.21 ± 0.07	˂0.05
OPG, pg/ml	2.70 ± 0.73	2.49 ± 0.58	ns

*SDS* standard deviation score, *BMI* body mass index, *HRT* hormonal replacement therapy, *GH* growth hormone, *IGF-1* Insulin Growth Factor 1; *Ca* calcium, *P* phosphorus, *bALP* bone alkaline phosphatase, *PTH* parathyroid hormone, *BMAD* bone mineral apparent density, *DKK-1* Dickkopf-1, *RANKL* receptor activator of NF-kB ligand, *OPG* osteoprotegerin

### Correlations of sclerostin and DKK-1 with anthropometric, bone metabolism markers, treatments, and BMAD

Table 2 shows the correlations between sclerostin and DKK-1 with anthropometric, bone metabolism markers, HRT, years of HRT therapy, rhGH therapy, and BMAD. In detail, sclerostin levels were positively related to calcium, phosphorous, and osteocalcin serum levels. Whereas sclerostin levels were negatively related to weight-SDS, BMI–SDS, Tanner stage, years of HRT therapy, 25-OH Vitamin D, and PTH levels as well as to lumbar BMAD-*Z*-score. Interestingly, also the therapy affects the levels of this cytokine, that are higher in patients not yet subjected to HRT, and lower in that patients who were out of GH treatment.

**Table 2:** Correlations with anthropometric, bone metabolism markers and instrumental data

	Sclerostin	DKK-1
Height-SDS	–	*r *= − 0.231, *p *= 0.01*
Weight-SDS	*r *= − 0.222, *p *= 0.019	*r *= 0.634, *p *= 0.0001*
BMI–SDS	*r *= − 0.262, *p *= 0.005	*r *= 0.648, *p *= 0.0001*
Tanner stage (I/II/III/IV/V)	*r *= − 0.478, *p *= 0.0001	–
HRT (yes/no)	*r *= − 0.305, *p *= 0.001	–
Years of HRT therapy	r **= ** − 0.603; p < 0.012*	
rhGH therapy (yes/no)	*r *= 0.571, *p *= 0.0001	*r *= 0.235, *p *= 0.01*
Ca, mg/dl	*r *= 0.536, *p *= 0.0001	
P, mg/dl	*r *= 0.356, *p *= 0.0001*	*r *= 0.797, *p *= 0.01
bALP, μg/L	–	–
25-OH vitamin D	*r *= − 0.340, *p *= 0.0001	*r *= − 0.616, *p *= 0.0001*
PTH, pg/ml	*r *= − 0.609, *p *= 0.0001	*r *= − 0.262, *p *= 0.005*
Osteocalcin, ng/ml	*r *= 0.841, *p *= 0.005	–
Sclerostin, pmol/l	–	–
DKK-1, pg/ml	–	–
RANK-L, pmol/l	*r *= 0.234, *p *= 0.013	*r *= 0.617, *p *= 0.0001*
OPG, pg/ml	*r *= 0.378, *p *= 0.0001	*r *= 0.229, *p *= 0.01*
Lumbar BMAD-*Z*-score	*r *= -0.548, *p *= 0.0001	*r *= -0.193, *p *= 0.042*

*SDS* standard deviation score, *BMI* body mass index, *HRT* hormonal replacement therapy, *GH* growth hormone, *Ca* calcium, *P* phosphorus, *bALP* bone alkaline phosphatase, *PTH* parathyroid hormone, *BMAD* bone mineral apparent density, *DKK-1* Dickkopf-1, *RANKL* receptor activator of NF-kB ligand, *OPG* osteoprotegerin

*Adjustment for age

Differently, DKK-1 levels were positively related to weight-SDS, as well as to phosphorous and rhGH therapy. DKK-1 serum levels were also negatively related to 25-OH Vitamin D and PTH levels as well as with lumbar BMAD-*Z*-score.

Furthermore, sclerostin serum levels positively correlated with the increased RANKL levels of TS patients, whereas DKK-1 levels correlated with both RANKL and OPG levels.

### Multiple linear regression

Multiple linear regression analyses were performed to explore the factors affecting sclerostin and DKK-1 levels in TS patients. Multiple linear regression analysis for sclerostin as dependent variable showed that HRT, years of HRT, bALP, PTH, osteocalcin, 25-OH Vitamin D, lumbar BMD *Z*-score, and RANKL were the most important predictors in TS subjects (Table 3). Whereas multiple linear regression analysis for DKK-1 as dependent variable demonstrated that PTH, 25-OH Vitamin D levels, lumbar BMAD *Z*-score and RANKL were the most important predictors in TS subjects (Table 3).

**Table 3:** Multiple linear regression

Dependent variable	Independent variable	*β*	*p*	*r*
Sclerostin			0.0001	0.996
	HRT	0.205	0.0001	
	Years of HRT	0.064	0.0001	
	bALP	0.246	0.0001	
	PTH	− 0.360	0.0001	
	Osteocalcin	0.868	0.0001	
	25-OH vitamin D	0.395	0.0001	
	Lumbar BMAD-*Z*-score	0.191	0.001	
	RANKL	− 0.274	0.0001	
DKK-1			0.0001	0.946
	PTH	0.829	0.0001	
	25-OH vitamin D	0.484	0.0001	
	Lumbar BMAD-Z-score	** − **0.693	0.0001	
	RANKL	0.736	0.0001	

*HRT* hormonal replacement therapy, *bALP* bone alkaline phosphatase, *PTH* parathyroid hormone, *BMAD* bone mineral apparent density, *DKK-1* Dickkopf-1, *RANKL* receptor activator of NF-kB ligand

## Discussion

The present study analyzed the circulating sclerostin and DKK-1 levels, as markers of possible impairment of cells of the osteoblastic lineage, in skeletal fragility associated with TS. Our results displayed that TS patients have higher levels of these two inhibitors of Wnt signaling with respect to the controls. Furthermore, we did not measure an altered percentage of OCPs comparing TS subjects and controls. The significant increase of sclerostin and DKK-1 in TS subjects supports the altered lumbar BMAD-Z-score that we found in our study population. Furthermore, TS patients displayed significant reduced levels of bone remodeling markers, such as bALP, 25-OH Vitamin D, and osteocalcin. Consistently, sclerostin and DKK-1 are related with lumbar spine BMAD-*Z*-score and the above-mentioned bone remodeling markers.

Interestingly, sclerostin levels were indirectly related with HRT. This finding represented an important issue, because is still debated the age of beginning HRT in TS girls. Estrogen replacement therapy is essential for the induction of puberty and for the maintenance of bone health in TS as well as in other disorders of sexual development [21, 22]. Estrogens regulate epiphysis closure and decrease the growth; however, low estrogen amounts do not alter the effects of rhGH on final height [23]. Furthermore, in childhood the combined therapy with ultra-low-dose estrogen and rhGH may favor the growth [24, 25], and may offer other benefits [26]. Moreover, continuous estrogen therapy improves BMD in TS patients, and the best effects on BMD are negatively related to the starting age of adult-dose estrogen treatment [27]. A recent paper reported that the starting age of estrogen replacement therapy, and the time between the starting ages of estrogen replacement therapy and estrogen–progestin therapy negatively and independently correlated with BMAD, but not with volumetric bone mineral density, after adjustment for BMI and age [28]. Lumbar BMD negatively correlated with the starting age of estrogen–progestin therapy independent of BMI and age. Thus, the authors concluded that an early start of HRT, mainly estrogen–progestin therapy, is central to reach better lumbar BMD in TS young adults [28]. Interestingly, in our TS population, DKK-1 levels were related with BMI–SDS. This is an interesting issue as DKK-1 promotes adipocyte differentiation [29]. It is known that both sclerostin and DKK-1 can affect the expression of RANKL and OPG, pro- and anti-osteoclastogenic cytokines, respectively, in the cells of the osteoblastic lineage [13]. Both sclerostin and DKK-1 were related with the high RANKL and normal OPG levels in TS patients, suggesting that in this syndrome these molecules may directly affect osteoblast activity and indirectly osteoclastogenesis. These findings further confirm our previous results which demonstrated an enhanced osteoclastogenesis in girls and young women with TS [11]. Other researchers have reported a co-regulation between RANKL and DKK-1 expression in different diseases, such as prostate cancer [30], osteosarcoma [31], and in children with 21-hydroxylase deficiency on chronic glucocorticoid treatment [32]. This was also observed in in vitro studies using murine mesenchymal stem cells [33]. Sclerostin levels has also been related to RANKL in pathological conditions, including multiple myeloma [34], and rheumatoid arthritis [35].

With regard to rhGH treatment and its correlation with the levels of Wnt inhibitors, it is important to remember that insulin-like growth factor (IGF-1), which is secreted from hepatocytes by GH, has an anabolic effect on bone growth by suppressing osteoblast apoptosis and enhancing osteoblastogenesis through a stabilization of the Wnt/*β*-catenin pathway [36]. In addition, IGF-1 reduces bone resorption through the OPG and RANKL system [37].

Unfortunately, we did not find statistically significant differences between TS patients and controls for the percentage of OCPs. Previously, it has been reported that these cells are more numerous and more immature in patients affected by fragility fractures than in osteoporotic patients without fractures [18], and it has been hypothesized that it could be due to a decreased homing ability in the skeleton. In this same study, it is reported that the treatment of patients with teriparatide enhanced the maturation of OCPs, but did not alter their percentage in the circulation [18]. However, it is important to specify that as summarized by Feehan et al. there is little agreement among studies with regard to the characterization of OCPs, with individual research groups using different cellular markers and criteria to identify these cells [38]. This disagreement and contradiction have reduced the applicability to the current research and restricted the progress in this area.

Finally, multiple linear regression analysis highlighted as main predictors of sclerostin levels in TS patients bALP, PTH, 25-OH vitamin D, osteocalcin, lumbar-BMAD-Z-score, and RANKL. Differently, for DKK-1 serum levels the best predictors are PTH, 25-OH Vitamin D, lumbar-BMAD-Z-score, and RANKL.

In conclusion, our results demonstrated that TS patients show higher levels of sclerostin and DKK-1 with respect to the controls which can be related to HRT, and to reduced bone formation markers as well as the increased bone resorption activity.
